# Bispecific T cell engagers: an emerging therapy for management of hematologic malignancies

**DOI:** 10.1186/s13045-021-01084-4

**Published:** 2021-05-03

**Authors:** Zheng Tian, Ming Liu, Ya Zhang, Xin Wang

**Affiliations:** 1 Department of Hematology, Shandong Provincial Hospital, Cheeloo College of Medicine, Shandong University, Jinan, 250021 Shandong China; 2 Department of Hematology, Shandong Provincial Hospital Affiliated To Shandong University, Shandong First Medical University, No.324, Jingwu Road, Jinan, 250021 Shandong China; 3 School of Medicine, Shandong University, Jinan, 250012 Shandong China; 4 Shandong Provincial Engineering Research Center of Lymphoma, Jinan, 250021 Shandong China; 5 Branch of National Clinical Research Center for Hematologic Diseases, Jinan, 250021 Shandong China; 6 National Clinical Research Center for Hematologic Diseases, The First Affiliated Hospital of Soochow University, Suzhou, 251006 China

**Keywords:** Bispecific T cell engager, Bispecific antibody, Cancer immunotherapy, Hematologic malignancy

## Abstract

Harnessing the power of immune cells, especially T cells, to enhance anti-tumor activities has become a promising strategy in clinical management of hematologic malignancies. The emerging bispecific antibodies (BsAbs), which recruit T cells to tumor cells, exemplified by bispecific T cell engagers (BiTEs), have facilitated the development of tumor immunotherapy. Here we discussed the advances and challenges in BiTE therapy developed for the treatment of hematologic malignancies. Blinatumomab, the first BiTE approved for the treatment of acute lymphocytic leukemia (ALL), is appreciated for its high efficacy and safety. Recent studies have focused on improving the efficacy of BiTEs by optimizing treatment regimens and refining the molecular structures of BiTEs. A considerable number of bispecific T cell-recruiting antibodies which are potentially effective in hematologic malignancies have been derived from BiTEs. The elucidation of mechanisms of BiTE action and neonatal techniques used for the construction of BsAbs can improve the treatment of hematological malignancies. This review summarized the features of bispecific T cell-recruiting antibodies for the treatment of hematologic malignancies with special focus on preclinical experiments and clinical studies.

## Background

Over the past few decades, bispecific antibodies (BsAbs) have been developed rapidly for the treatment of hematologic malignancies. There are more than 100 formats for BsAbs, of which bispecific T cell engagers (BiTEs) are well-designed formats, and novel structures of BsAbs are emerging constantly [1]. The concept of BsAbs first appeared in the early 1960s, with the first example constructed in 1985 [2]. BiTE is the BsAb designed to target CD3 and tumor-specific antigens simultaneously and promote the cytotoxicity of T cells. Since Blinatumomab, a canonical CD3/CD19 BiTE, was approved by the United States Food and Drug Administration (FDA) in December 2014 for adult Philadelphia chromosome negative (Ph-) relapsed or refractory (R/R) B cell progenitor acute lymphoblastic leukemia (B-ALL), BiTEs for the management of hematologic malignancies have been developed rapidly [3]. This review summarized the current research status of BiTEs for the treatment of hematologic malignancies. Many bispecific T cell-recruiting antibodies with novel structures have been derived from BiTEs. Some bispecific T cell-recruiting antibodies have been approved for the treatment of hematologic malignancies and multiple promising drugs are currently in clinical trials. In order to maximize the therapeutic effects of bispecific T cell-recruiting antibodies, research issues including the response rates, the recommended doses and adverse events need to be discussed.

## Structures of BsAbs

BsAbs are divided into three categories according to their targets: (i) antibodies targeting two different tumor antigens; (ii) antibodies targeting one tumor antigen and one immune-related molecule; (iii) antibodies targeting two immune-related molecules. BiTEs belong to the second category because one BiTE molecule usually targets one CD3 molecule and one tumor antigen simultaneously.

BsAbs are developed on the basis of monoclonal antibodies. In the early days of BsAb development, BsAbs were produced by the reduction and reoxidation of hinged cysteine in monoclonal antibodies [4]. At the present time, according to the structures of BsAbs, BsAbs are divided into two categories: the immunoglobulin G (IgG)-based antibodies and the variable fragment (Fv)-based antibodies [5].

BsAbs based on the IgG structure display a similar structure to native antibodies. The major method of producing IgG-based BsAbs is recombing half-molecules from heterogenous parental antibodies. New techniques of recombining functional half-molecules to produce IgG-based BsAbs include, but are not limited to orthogonal Fab interface, DuoBody, XmAb, CrossMab, and knobs-into-holes (KiH) [6–10]. Concerning the selection of IgG subclass, IgG2 and IgG4 are suitable options because IgG1-based antibodies can cause the elimination of activated T cells [11]. Duobody developed by Genmab is the platform which enables production of BsAbs by exchanging half-molecules from different parental IgGs. The mutation in the constant region of the heavy chain (C_H_) can recognize the heterologous half-molecule and promote the procedure of heterodimerization. KiH technology developed by Roche also enables production of antibodies through exchanging half-molecules. Knobs and holes mean mutations on C_H_3 domains which can promote heterodimerization between half-molecules. Based on KiH technology, Roche developed the CrossMab platform by exchanging the C_H_1 and the constant region of the light chain (C_L_) of one parental antibody. This technique can solve the problem of light chain mismatching. XmAb technology developed by Xencor also enables production of BsAbs nearly identical to natural antibodies. Compared with Fv-based BsAbs, IgG-based antibodies have longer half-lives in vivo because they are larger in size and are hard to be cleared by the kidney. The solubility and stability of BsAbs are also improved for the presence of the fragment crystallizable (Fc) domains [12]. Fc domains of BsAbs can recruit natural killer (NK) cells and macrophages to induce antibody-dependent cell-mediated cytotoxicity (ADCC) and complement-dependent cytotoxicity (CDC) [13]. However, the disadvantages of IgG-based BsAbs are also notable. The permeability of IgG-based BsAbs to tumor tissues is lower than Fv-based BsAbs because of the increased molecular weight. And the production of IgG-based antibodies requires more complex techniques.

**Fig. 1:**
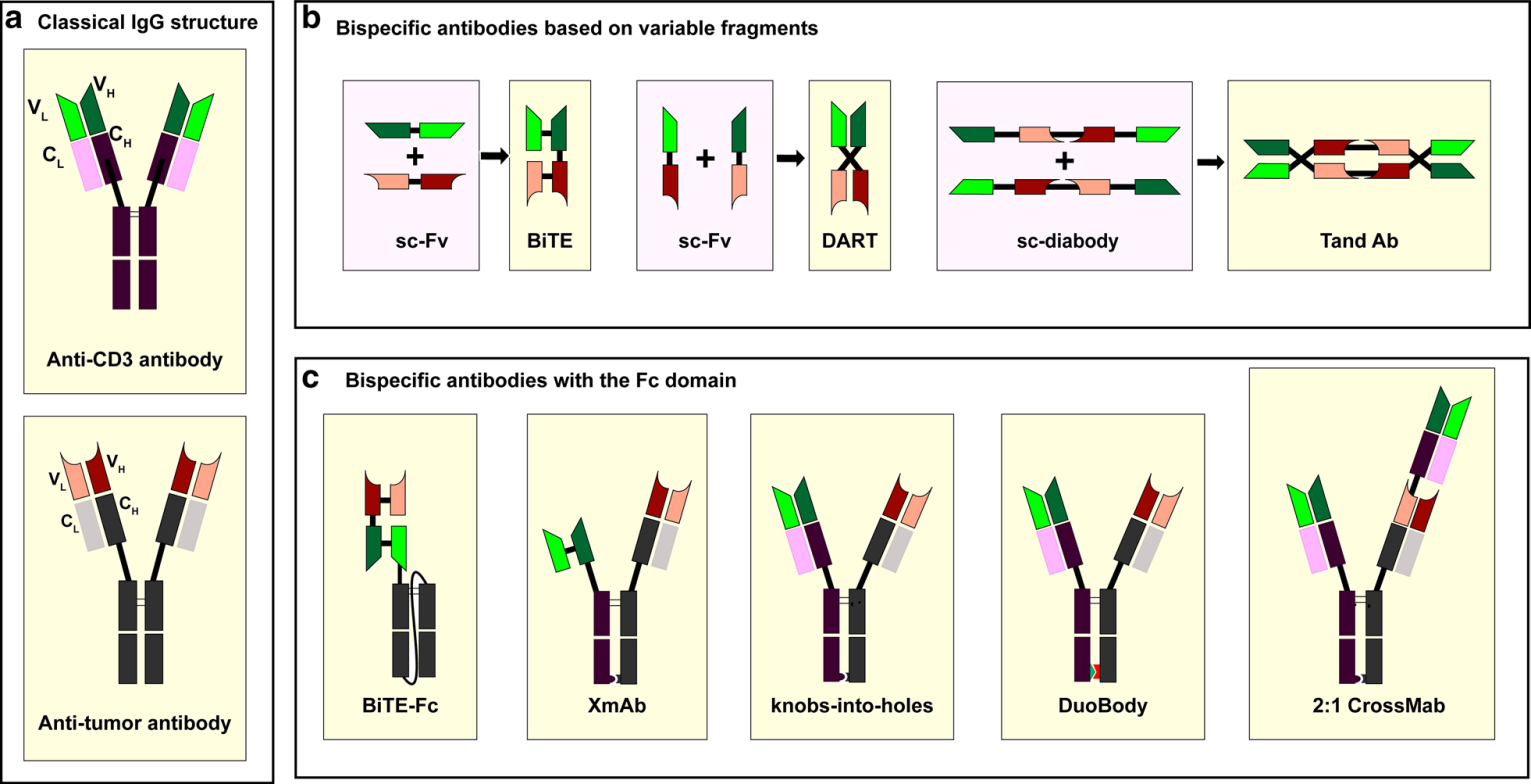
Structures of bispecific T cell-recruiting antibodies. A bispecific T cell engager (BiTE) consists of two single-chain variable fragments (scFvs); a dual-affinity retargeting antibody (DART) consists of two engineered scFvs whose V_H_ exchanged with the other one; a TandAb consists of two single-chain diabodies with four variable domains; a XmAb consists of one scFv, one Fab fragment and one hetero-Fc domain; a 2:1 Crossmab contains two tumor antigen binders and one CD3 binder; “knob in hole” technique and duobody technique enable production of bispecific antibodies with similar structures to natural IgG

The Fv-based BsAbs usually consist of single-chain variable fragments (scFvs) simply. Due to their short half-lives, continuous infusion is required, which has restricted their promotion [12]. The BiTE technique is the prime platform to produce Fv-based BsAbs. Except for BiTE, single-chain diabody, dual-affinity retargeting antibody (DART), and tandem diabody (TandAb) are also platforms used for producing Fv-based BsAbs [14–16]. The BiTE molecule developed by Micromet is the antibody consisting of two scFvs connected by a short peptide linker. The scFv is an antibody fragment produced by fusing one variable region of the heavy chain (V_H_) and one variable region of the light chain (V_L_) artificially. The DART molecule developed by MacroGenics is the antibody consisting of two engineered heterogenous scFvs which have exchanged their V_H_ regions. The TandAb molecule developed by Affimed is the antibody consisting of two single-chain diabodies which contain four variable domains, respectively. One TandAb molecule is constructed of two binding sites for CD3 and two binding sites for tumor antigens [17]. The BsAbs mentioned in this passage and their structures are illustrated in Table 1 and Fig. 1.

**Table 1:** Bispecific T cell-recruiting antibodies for the treatment of hematologic malignancies

Disease	Target	Name	Antibody format
AML	CD123-CD3	MGD006	DART
		XmAb14045	XmAb
	CD33-CD3	AMG 330	BiTE
		AMV 564	TandAb
	FLT3-CD3	7370	BiTE
	CLEC12A-CD3	MCLA-117	Biclonics
	WT1-CD3	ESK1-BiTE	BiTE
ALL	CD19-CD3	Blinatumomab	BiTE
		AFM11	TandAb
MM	BCMA-CD3	AMG420	BiTE
		AMG701	BiTE-Fc
	GPRC5D-CD3	Talquetamab	DuoBody
	CD38-CD3	AMG424	XmAb
		Bi38-3	BiTE
	FCRL5-CD3	anti-FcRH5/CD3 TDB	Knobs-into-holes
NHL	CD19-CD3	Blinatumomab	BiTE
	CD20-CD3	REGN1979	Veloci-Bi platform
		Mosunetuzumab	Knobs-into-holes
		RG6026	2:1 CrossMab
MDS	CD33-CD3	AMV564	TandAb
	CD123-CD3	MGD006	DART

## Mechanisms of BiTE action

Different from natural antibodies, BiTEs can redirect T cells to specific tumor antigens and activate T cells directly. Natural antibodies are unable to recruit T cells directly because T cells lack Fcγ receptors [18]. The BiTE molecule usually targets one tumor antigen and one CD3 molecule simultaneously. The CD3 molecule non-covalently associates with the T cell receptor (TCR) and participates in antigen-specific signals transduction which can induce the activation of T cells. Activated T cells express high levels of CD69 and CD25 which promote the proliferation of T cells [19]. BiTE therapy can be a strategy to activate exhausted T cells induced by long-term exposure to tumor antigens. Some features of T cell activation induced by BiTEs have been observed. Firstly, the tumor cell plays an indispensable role in the T cell activation induced by the BiTE. Secondly, T cells can be activated without costimulatory signals such as CD28 and interleukin (IL)-2 [20]. This feature is attributed to memory T cells which play an important role in the reaction to BiTEs [21]. Another explanation is that immunological synapses between T cells and tumor cells can assemble TCRs and amplify first signals [20].

**Fig. 2:**
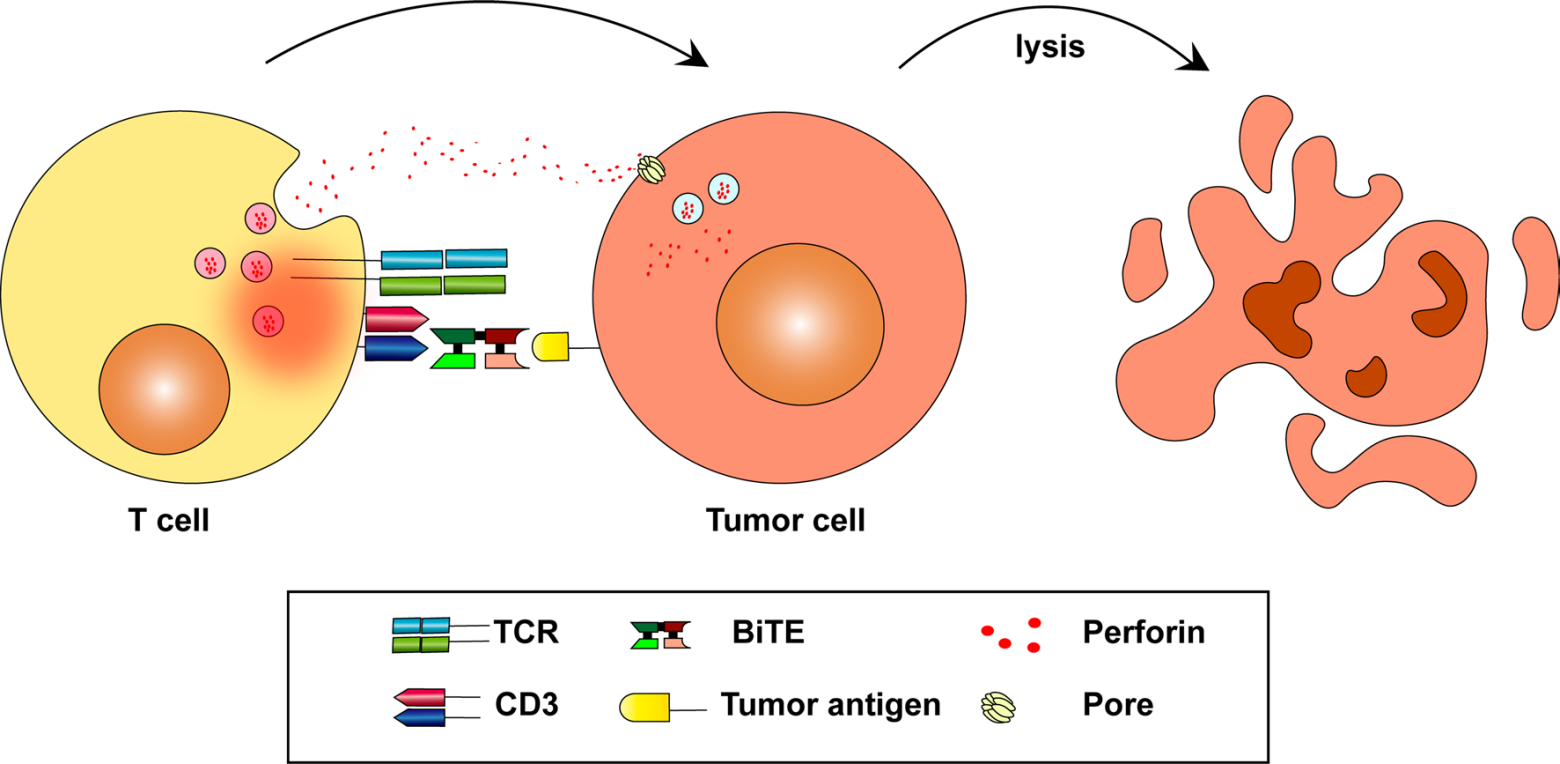
Mechanisms of tumor cell lysis mediated by the BiTEs. BiTEs can redirect T cells to tumor cells and active T cells. Activated T cells release perforin and other granzymes through immunological synapses. These cytolytic proteins can form endosomes in tumor cells and lyse tumor cells ultimately

Immunological synapses between T cells and tumor cells are also essential to the BiTE-mediated tumor lysis [22–25]. Activated T cells secrete perforin and other granzymes through immunological synapses. These cytolytic proteins can form pores on cancer cell membrane. During the process of membrane self-repair, perforin, and other granzymes are endocytosed by cancer cells and then form endosomes. Perforin inside endosomes can form pores on the endosomal membrane and cause the release of granzymes inside targeted cells, then cancer cells are lysed (Fig. 2) [26, 27].

### Mechanisms of tumor escape

Although BiTEs have been proved to be efficient in many relapsed or refractory hematological malignancies, a subset of hematological malignancy patients still have no response to BiTEs. In order to improve the efficacy of BiTEs, further studies on tumor escape should be implemented. The suppression of immune system, especially suppression of T cells, is an important reason for tumor escape [28]. The relationship between BiTE resistance and programmed cell death protein 1/ programmed death-ligand 1 (PD-1/PD-L1) axis has been demonstrated [29]. The expression level of PD-L1 increased in blinatumomab-resistant patients, which indicated the potential efficacy of BiTE therapy in combination with PD-1/PD-L1 inhibitors [30]. AMG 330, a CD33/CD3 BiTE, caused elevated PD-L1 expression on AML cells. And PD-1/PD-L1 blockade therapy enhanced the anti-tumor efficacy of AMG 330 [31]. A large amount of clinical trials focusing on combination therapy with bispecific T cell-recruiting antibodies and PD-1/PD-L1 inhibitors are ongoing. An antibody which targets PD-1, CD3 and CD33 simultaneously has been developed and proved to be efficient in treating acute myeloid leukemia (AML) in preclinical experiments [32].

Loss of target antigen expression is another explanation for tumor escape. It was reported that 8% R/R acute lymphoblastic leukemia (ALL) cases after blinatumomab therapy were CD19 negative [33, 34]. It means other therapies targeting CD19 are potentially effective in most patients after blinatumomab therapy. However, understanding the mechanism of CD19 downregulation can improve the prognosis of CD19-relapsed patients. Antigenic shift is not the only reason for the downregulation of target antigens. It can also be attributed to the disrupted trafficking of the target antigens [35].

### Acute lymphoblastic leukemia

**Fig. 3:**
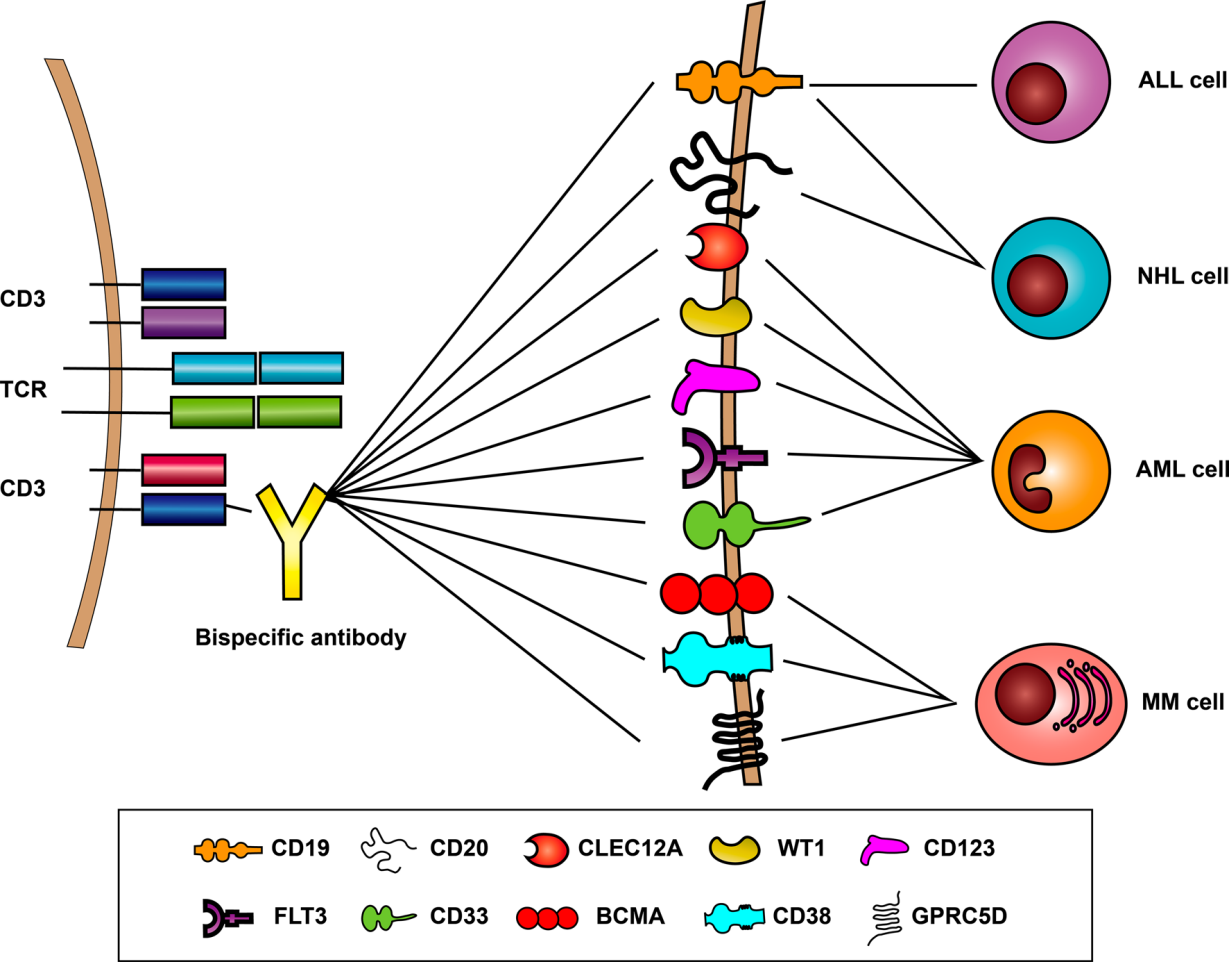
Targets of bispecific B cell-recruiting antibodies. CD19 and CD20 are targets for treatment of NHL; CD19 is the target for treatment of acute lymphocytic leukemia; CD123, CD33, CLEC12A, WT1, and FLT3 are targets for treatment of acute myeloid leukemia; BCMA, CD38, and GPRC5D are targets for treatment of multiple myeloma

ALL is characterized by the proliferation of a huge number of immature lymphocytes in different tissues. R/R ALL patients used to have poor clinical outcomes even after heavy salvage chemotherapy and hematopoietic stem cell transplantation (HSCT) [36, 37]. However, in recent years, new targeted drugs have been developed as remedies for ALL. These drugs include BsAbs, CAR-T cells, anti-CD20 monoclonal antibodies, and tyrosine kinase inhibitors [38–40]. BsAbs targeting CD19 and CD3 for the treatment of ALL have become a subject undergoing intense study recently because CD19 is overexpressed in ALL cells (Fig. 3) [5].

### Blinatumomab

Blinatumomab (MT103) is a BiTE that consists of two scFvs which can combine with CD19 and CD3 separately. The anti-CD19 domain and anti-CD3 domain of blinatumomab are connected with a short glycine–serine linker. The flexible structure of blinatumomab enables blinatumomab to bind T cells and tumor cells efficiently [41]. Blinatumomab has been proved to be an efficient drug in ALL and non-Hodgkin lymphoma (NHL) patients. Blinatumomab could activate cytotoxic T cells and induce regression of NHL cells at the concentration as low as 0.06 mg/m2/day [42, 43]. The FDA has approved blinatumomab for the treatment of R/R Philadelphia chromosome-negative (Ph-) B-ALL in 2014 and R/R Philadelphia chromosome-positive (Ph +) B-ALL in 2017. Blinatumomab is also conducive to deleting minimal residual disease (MRD) in AML [44]. MRD is the status of persistent detectable leukemia cells after complete remission assessed by traditional pathological examinations. MRD is predictive of poor patient prognosis and the deletion of MRD is beneficial to patients [45]. Blinatumomab was approved for the treatment of MRD in B-ALL patients in 2018 and it is the first FDA-approved treatment for MRD.

### Efficacy of blinatumomab

Blinatumomab is an effective therapy for R/R Ph- B-ALL. In a phase II study which included primary R/R Ph- ALL patients, 43% of the patients achieved complete remission (CR) (33%) or complete remission with partial hematologic recovery in peripheral blood (CRh) (10%) after two cycles of blinatumomab treatment [46]. More detailed information about clinical trials of blinatumomab and some drugs mentioned in this passage are listed in Table 2. In patients who relapsed after allogeneic hematopoietic stem cell transplantation (allo-HSCT), the CR/CRh rate after two cycles of blinatumomab treatment was 45%. These data have corroborated that blinatumomab is significantly effective in aggressive ALL (NCT01466179) [47]. In a phase II study which included 108 Ph- B-ALL patients in CR with MRD, 85 patients reached MRD negativity after one cycle of blinatumomab treatment at the dose of 15 µg/m^2^/day. And blinatumomab administered in first CR is associated with the long-term survival of patients. Thus, early administration of blinatumomab is beneficial to B-ALL patients with MRD (NCT01207388) [48]. Compared with standard chemotherapy, blinatumomab was associated with a higher rate of CR and longer overall survival [49]. Moreover, administration of blinatumomab before allogeneic stem cell transplantation improved the rate of CR, which indicated that blinatumomab could be a bridge to allogeneic stem cell transplantation (NCT02013167) [50].

**Table 2:** Clinical trials of bispecific antibodies in hematologic malignancies

Drug	Disease	Target	Phase	Identifier	Dose	Number	Response	Adverse events
Blinatumomab	R/R Ph + ALL	CD19/CD3	II	NCT02000427	9 mg/day (first 7 days) 28 mg/day (following days)	*n* = 45	CR/CRh rate = 35.6%	AE(common): pyrexia (58%), febrile neutropenia (40%), and headache (31%)
								AE(grade ≥ 3): febrile neutropenia (27%), thrombocytopenia (22%), and anemia (16%)
	R/R B-ALL	CD19/CD3	II	NCT01466179	9 μg/day (first 7 days)	*n* = 189	CR/CRh rate = 43%	AE (common): pyrexia (113, 60%), headache (34%), febrile neutropenia (28%), peripheral oedema (26%), nausea (24%), hypokalaemia (24%), constipation (21%), anemia (20%)
					28 μg/day (following days)			AE (grade ≥ 3): febrile neutropenia (25%), neutropenia (16%), anemia (14%)
	R/R NHL	CD19/CD3	I	Not applicable	5 μg/m^2^/d (week 1)	*n* = 9	ORR = 56%	AE (common): pyrexia (81.8%), fatigue (54.5%), constipation (36.4%), headache (36.4%), tremor (36.4%), and weight increase (36.4%)
					15 μg/m^2^/d (week 2)			
					60 μg/m^2^/d (following days)			
MGD006	R/R AML	CD123/CD3	I/II	NCT02152956	500 ng/kg/day	*n* = 30	CR/CRh rate = 26.7%	AE (common): CRS (mild to moderate signs/symptoms) (81%),
							CR/CRh/CRi rate = 30.0%	Grade 3 events: (8%)
XmAb14045	R/R AML	CD123/CD3	I	NCT02730312	1.3 µg/kg/week and 2.3 µg/kg/week	*n* = 63	CR/CRi rate = 23%	AE (common): fatigue (31%), febrile neutropenia (30%), peripheral edema (30%), cough (23%), elevated hepatic transaminases (19%), pneumonia (17%), stomatitis (14%), hyperglycemia (13%), sepsis (11%)
AMG 330	R/R AML	CD33-CD3	I	NCT02520427	0.5–720 µg/day	*n* = 42	ORR = 19%	AE (common): cytokine release syndrome (67%), nausea (20%)
AMG420	R/R MM	BCMA/CD3	I	NCT02514239	400 µg/d recommended	*n* = 42	ORR = 31%	AE (common): infections, polyneuropathy
REGN1979	R/R FL (Gr 1-3a)	CD20/CD3	I	Not applicable	5-320 mg/week	*n* = 14	ORR = 93%	AE (common): pyrexia, CRS, chills, infections and infestations, fatigue, increased C-reactive protein, anemia
	R/R DLBCL				5-12 mg/week	*n* = 11	ORR = 18.2%	
					18–40 mg/week	*n* = 11	ORR = 54.5%	
					80 mg/week	*n* = 3	ORR = 100%	
					160 mg/week	*n* = 3	ORR = 33.3%	
RG6026	R/R B-NHL	CD20/CD3	I	NCT03625037	300–1800 µg	*n* = 24	ORR = 8/24	AE (common): pyrexia, neutropenia and cytokine release syndrome
	R/R FL				300–1800 µg	*n* = 5	ORR = 3/5	
Mosunetuzumab	Indolent NHL	CD20/CD3	I/Ib	NCT02500407; GO29781	inferior to 1/2/60 mg (Cycle 1 Day 1, 8, and 15)	*n* = 64	ORR = 64.1%	AE(common):CRS(28.4%),
							CRR42.2%	headache (14.7%), insomnia (10.1%), dizziness (9.2%)
	Aggressive NHL					*n* = 119	ORR34.7%	
							CRR = 18.6%	

Blinatumomab is also a treatment option for Ph + ALL patients. The prognosis of Ph + ALL patients has been greatly improved since the appearance of tyrosine kinase inhibitors (TKIs) exemplified by imatinib. However, R/R Ph + ALL patients who are resistant to imatinib therapy still lack access to effective therapies. And blinatumomab is a feasible solution to this problem [51]. Blinatumomab has more advantages than chemotherapy, including the increased rate of CR/CRh and longer overall survival (OS) [52]. In a phase II study which included Ph + ALL patients intolerant or refractory to imatinib, 36% of study subjects reached CR/CRh after two cycles of blinatumomab monotherapy (NCT02000427) [53]. Another phase II trial aimed to evaluate the efficacy of dasatinib–blinatumomab therapy in newly diagnosed Ph + ALL patients. 98% of the patients included in this trial achieved CR after the dasatinib induction treatment and the following blinatumomab cycles at the dose of 28 μg/day. Fifteen patients were MRD-positive after the dasatinib induction treatment. ABL1 mutations were detected in 7 of 15 MRD-positive patients. However, blinatumomab eliminated all the mutations related to MRD in this trial, which proved that blinatumomab had an advantage in deleting MRD cells with ABL1 mutations [44].

Meanwhile, the efficacy of blinatumomab against NHL has been evaluated by a series of clinical trials. In 2011, Blinatumomab was proved to be efficient in R/R diffuse large B cell lymphoma (DLBCL) patients and the objective response rate (ORR) reached 56%. In a phase I clinical trial which included R/R NHL patients, the maximum tolerated dose (MTD) of blinatumomab was determined to be 60 μg/m^2^ per day. Among patients treated at the MTD, the ORR reached 69% (FL, 80%; MCL, 71%; DLBCL, 55%) and the CR/CRu rate reached 37% [54]. In another phase II study which included R/R DLBCL patients (*n* = 25), the ORR and CR rate were 43% (9/21) and 19% (4/21) respectively at the dose of 112 μg per day [55].

### Administration of blinatumomab

The optimal dosing regimen of blinatumomab to maximize therapeutic effects has been determined. The molecular weight of blinatumomab is low (~ 55 kDa) because the BiTE molecule only consists of two scFvs. And the estimated mean (standard deviation) of elimination half-life of blinatumomab is 2.11 (1.42) h. Because of its short half-life, blinatumomab is usually administered by continuous intravenous infusion to maintain its therapeutic concentration [56]. The recommended dose of blinatumomab was determined in a phase II study (NCT01209286) and the regimen is described below. Each cycle lasts 6 weeks. During the first cycle, blinatumomab should be administered at the dose of 9 μg/day in the first week. Then the dose rises to 28 μg/day during the following 3 weeks. There is an interval for 2 weeks at the end of the first cycle. During the following cycles, each cycle includes continuous blinatumomab administration at the dose of 28 μg/day for 4 weeks and an interval for 2 weeks [57].

### Adverse events of blinatumomab

A number of adverse events have been observed during blinatumomab cycles. In a phase II study which included R/R B-ALL patients, the common adverse events during blinatumomab therapy included pyrexia (81%), fatigue (50%), headache (47%), tremor (36%), and leukopenia (19%). Most of the adverse events occurred during the first cycle of administration [57]. In a study including adult Ph- R/R ALL patients in Korean, the most common side effects included infection (50%), neurological adverse events (36%), and cytokine release syndrome (CRS) (20%; CRS of grade 3 or higher, 4%) [58]. In another trial including Ph + ALL patients, the most common adverse events of grade 3 or higher involved neutropenia (27%), thrombocytopenia (22%), and anemia (16%) [52]. Adverse effects were compared between the blinatumomab group and the chemotherapy group in a clinical trial which included advanced ALL patients. Although patients in the blinatumomab group suffered more adverse events, the rate of severe adverse effects in the blinatumomab group was lower than the rate in the chemotherapy group [68]. Compared with CAR-T therapy, blinatumomab is associated with a lower incidence of CRS, which is the advantage of blinatumomab [34].

Severe CRS and neurological adverse events are the main reasons for the interruption of blinatumomab therapy. CRS is caused by the release of a large number of cytokines and the following systemic inflammation. The clinical manifestations of CRS include high fever, skin rash, vomiting, and nausea [59]. This problem can be attributed to abnormal activation of effector T cells and macrophages induced by blinatumomab. Activated T cells release high levels of IFN-γ and other cytokines, which can induce macrophages to release high levels of IL-6 and IL-10 subsequently. Severe patients even suffer the problem of hemophagocytic lymphohistiocytosis (HLH) [60]. IL-6 is the center of this pathological process. Other cytokines including TNF, IL-2, GM-CSF, and IL-5 also participate in this process [61, 62]. Severe CRS can be prevented by the administration of dexamethasone beforehand and stepwise dosing [57, 58, 63]. Tocilizumab, an anti-IL-6 receptor monoclonal antibody, is effective in patients with HLH [60].

The neurological adverse events can be attributed to the redistribution of activated T cells. Activated T cells induced by blinatumomab attach to cerebral vessels and move to cerebrospinal fluid. Sedimentation of T cells causes microcirculation dysfunction and local ischemia which ultimately result in neurological symptoms. Patients with diminished B/T cell ratios are more likely to suffer neurological adverse events [64]. Although neurological adverse events can occur in patients with CRS, there is still no evidence that neurological adverse events are associated with the release of cytokines [65]. The neurological symptoms can be controlled after the withdrawal of blinatumomab treatment [57]. Severe neurological events can be prevented by administration of steroids in advance and close clinical monitoring [66].

### AFM11

Although blinatumomab is a promising drug to defeat ALL, this antibody is still limited in clinical practice. Firstly, blinatumomab requires persistent administration due to its short half-life, which limits the generalizing of blinatumomab. Secondly, only less than half of the patients with R/R ALL had a partial or complete response to blinatumomab therapy whereas other R/R ALL patients lacked effective treatments. A new drug with higher efficacy and a longer half-life is in demand.

AFM11 is a TandAb with a longer half-life and better affinity to both CD3 and CD19. The efficacy of AFM11 has been demonstrated in preclinical studies. In vitro experiments have proved that AFM11 mediated the CD19 specific cytotoxic effect. AFM11 could activate T cells and promote apoptosis of leukemia cells in chronic lymphocytic leukemia (CLL) and small lymphocytic lymphoma. The cytotoxicity of AFM11 was stronger than blinatumomab in vitro [67]. AFM11 could also reinvigorate exhausted T cells induced by chemotherapy [68]. AFM11 was investigated in a phase I clinical trial which included patients with R/R B-ALL. However, the development of AFM11 was interrupted after one death caused by neurological adverse events.

### Acute myeloid leukemia

Acute myeloid leukemia (AML) is the most frequent acute leukemia in adults and the incidence increases with age [69]. AML is a highly heterogeneous disease caused by mutations in hematopoietic stem/progenitor cells. Multiple mechanisms including upregulation of Wnt signaling pathway result in abnormal proliferation and differentiation of bone marrow stem cell clones [70]. The treatment of AML is still a gigantic challenge in the field of hematologic oncology. Cytosine arabinoside and anthracyclines are the basis of AML therapy. About 40%-45% of young patients and 10%-20% of elderly patients can be cured in this way [71]. However, the cure rate for patients with R/R AML is lower than 10% [72]. Allo-HSCT used to be the single choice for R/R AML patients, but only a fraction of patients received this treatment [73]. Elderly patients rarely received this treatment because allo-HSCT was associated with poor prognosis and a high recurrence rate among them [74]. Immunotherapy which stimulates the immune system by targeting immune pathways has revolutionized the field of AML therapy. Gemtuzumab ozogamicin was approved as an antibody–drug conjugate for the treatment of AML in 2000, which has significantly promoted the development of other immunotherapies such as BsAbs [75]. Currently, a considerable number of BsAbs for the treatment of AML are in clinical trials and the members include BiTEs, DARTs, and TandAbs. Many tumor surface antigens are potential targets of BsAbs, such as CD123, CD33, FMS-like tyrosine kinase 3 (FLT3), C-type lectin domain family 12 member A (CLEC12A), and Wilms' tumor gene 1 (WT1) [76].

### CD123/CD3

CD123, also known as the interleukin 3 receptor alpha chain (IL-3Rα), is overexpressed in many hematological malignances, including AML, Hodgkin lymphoma (HL), and blastic plasmacytoid dendritic cell neoplasm [77]. CD123 is mainly overexpressed in CD34 + /CD38- AML cells [78]. And the overexpression of CD123 is predictive of inferior prognosis [79]. Several BsAbs targeting CD123 and CD3 simultaneously such as MGD006, XmAb14045, and JNJ-63709178 are currently in clinical trials for the treatment of AML. And the preliminary results of clinical trials focusing on MGD006 and XmAb14045 have been published.

### MGD006

MGD006 is a DART which targets CD123 and CD3 simultaneously [80]. Compared with BiTEs, it has better stability and manufacturability. It recruits T lymphocytes to CD123 + tumor cells, which induces T cell activation, T cell proliferation and CD123 + tumor cell lysis [81]. Currently, the safety and efficacy of MGD006 in AML patients has been demonstrated in a phase I/II clinical trial (NCT02152956). The CR/CRh rate was 26.7% and the total effective rate (CR/CRh/ complete remission with incomplete count recovery) was 30.0% among primary induction failure/early relapsed patients treated at the recommended phase II dose of 500 ng/kg/day. The median overall survival was 10.2 months and the 6- and 12-month survival rates were 75% and 50%, respectively, among patients who achieved CR/CRh [82]. MGD006 needs continuous infusion because the half-life of MGD006 is short [83]. Like other BsAbs, the most common adverse reaction to MGD006 is CRS. The trial is ongoing and the focus of the research is laid on primary induction failure/early relapsed AML.

### XmAb14045 (Vibecotamab)

XmAb14045 is an anti-CD123/CD3 BsAb produced by XmAb technique. Different from MGD006, XmAb14045 is intermittently administered because the half-life of XmAb14045 is extended. XmAb14045 has been investigated in a phase I clinical study since 2016. In part A of the study, 23% of the 64 R/R AML patients had complete responses to XmAb14045 monotherapy. One of them remained in remission 14 weeks after the initial treatment without stem cell transplantation. CRS was present in 77% of the R/R AML patients treated with XmAb1404. The optimal dosing regimen is being studied in part B of this clinical trial [77].

### CD33/CD3

CD33, a member of sialic acid-binding sialoadhesin receptors, is selectively expressed on AML cells [84]. It was reported that CD33 was expressed in 87.8% of 319 AML patients, thus CD33 could be an ideal therapeutic target for AML [85]. Since gemtuzumab ozogamicin was approved for treating AML, BsAbs targeting CD33 and CD3 have been developed rapidly. BsAbs including AMG330 (NCT02520427), AMG673 (NCT03224819), AMV564 (NCT03144245), and GEM333 (NCTT03516760) are currently in clinical trials.

### AMG330

AMG330 is a BiTE binding with CD33 and CD3 simultaneously. AMG330 can promote the proliferation and activation of T cells, which result in the lysis of CD33 + human AML cells [86]. AMG330 has been demonstrated to attack CD33 + myeloid derived suppressor cells (MDSCs) through T cell-mediated cytotoxicity [87]. An ongoing phase I clinical trial of AMG330 is investigating the safety and tolerated dose of this antibody (NCT02520427).

### AMV564

AMV564 is a TandAb containing two binding sites for CD3 and two binding sites for CD33 separately. This structure improves not only its binding affinity for target cells, but also its molecular weight. Because of the increased molecular weight, AMV564 is more difficult to be cleared by the kidney compared with AMG330, which gives it a longer half-life [88]. Phase I clinical trials of AMV564 in patients with AML, myelodysplastic syndromes (MDS), and solid tumors are currently ongoing. In a phase I clinical trial, R/R AML patients received increasing doses of intravenous injection of AMV564 for 14 days. It has been demonstrated that AMV564 was well tolerated, safe and selective. The annual meeting of the Cancer Immunotherapy Society (SITC) also reported that AMV564 could bolster anticancer immunity by depleting immunosuppressive MDSCs and promoting T cell activation.

### FLT3/CD3

FLT3 is a class III receptor tyrosine kinase that plays an important role in the proliferation of hematopoietic cells and lymphocytes. It regulates the survival and growth of hematopoietic stem cells, maturation of dendritic cells, and maintenance of regulatory T cell homeostasis [89]. Aberrant expression of FLT3 is closely related to the occurrence of AML and other malignant tumors. FLT3 is overexpressed in more than 70% of AML cases, so FTL3 is an effective target for AML treatment [90]. 7370 is an anti-FLT3/CD3 IgG-based BsAb with advantages of a long half-life and high affinity. 7370 could potently activate human T cells against FLT3 + AML cells in vivo. And 7370 was well tolerated in cynomolgus monkeys. Moreover, 7370 has shown potential clinical values in AML patients regardless of the FLT3 mutation status [91].

### CLEC12A/CD3

CLEC12A, also known as human C-type lectin like molecule-1 (CLL-1) or myeloid inhibitory C‐type lectin‐like receptor (MICL), is specifically expressed in AML progenitor cells and leukemia stem cells [92]. CLEC12A is overexpressed in 90–95% of new or recurrent AML cases, but it is rare in normal tissues. Thus, CLEC12A is a potentially effective target for AML therapy [93]. Supported by Biclonics platform, MCLA-117 is a full-length human bispecific IgG that specifically binds CLEC12A + AML cells and CD3 + T cells. Pieter Fokko Van Loo et al. demonstrated that MCLA-117 effectively recruited cytolytic T cells to attack tumor cells in 10 of 11 primary AML samples. MCLA-117 strongly induced killing of AML cells (23%-98%) at low effector-to-target ratios (1:3–1:97) through activating autologous bone marrow T cells in primary AML patient samples. Because MCLA-117 has the potential to selectively target CLEC12A + myeloid cells without affecting normal hematopoietic stem cells, it has the ability to restore normal hematopoietic function and prevent the hematotoxicity induced by MCLA-117 [94]. MCLA-117 is currently under a phase I clinical study (NCT03038230) which focuses on evaluating the efficacy and safety of MCLA-117 in adult patients with AML.

### WT1/CD3

WT1 is a tumor-associated antigen located on chromosome 11p13. WT1 plays an important role in the control of cell growth and differentiation [95]. WT1 is overexpressed in leukemia and many solid tumors, especially in AML samples. Therefore, WT1 is an ideal target for AML therapy [96]. A BiTE molecule derived from ESK1, a TCR-mimic monoclonal antibody, can specifically bind WT1 + cells from HLA-A*02:01 + AML cell lines and CD3 + T cells. Its efficacy of killing WT1 + AML cells has been proved by experiments in vitro and in mouse models [97].

### Multiple myeloma

Multiple myeloma (MM) is characterized by the proliferation of malignant plasma cells [98]. The median overall survival of MM patients has been improved to 5 years because of the introduction of new drugs [99]. Although the survival of patients is increasing with the continuous progress of therapeutic regimens, MM is still an incurable disease and almost all MM patients eventually relapsed [100]. Many bispecific T cell-recruiting antibodies for the treatment of MM have been developed rapidly in recent years. The ideal targets of BsAbs for treating MM include B cell maturation antigen (BCMA), G protein-coupled receptor 5D (GPRC5D), CD38, and Fc receptor-like 5 (FCRL5).

### BCMA/CD3

BCMA, a tumor necrosis factor receptor, is generally expressed on mature B cells. It plays a key role in the survival and proliferation of B cells, especially the long term survival of plasma cells [101]. The ligand of BCMA, a proliferation-inducing ligand, also participates in the progression of MM [102]. BCMA is expressed specifically on MM cells, which makes it an ideal target for the therapy of MM. Serum B cell maturation antigen (sBCMA) is produced by BCMA cleavage and is related to poor clinical outcomes [103]. The poor prognosis can be attributed to its interaction with BCMA signaling pathways in normal cells [104]. There have been many BCMA targeted therapies for the treatment of MM patients, such as BsAbs, CAR-T cells, and antibody–drug conjugates [102, 105, 106]. BiTEs targeting BCMA have been investigated in clinical trials and the preliminary results are promising.

### AMG 420

AMG 420 is a BiTE composed of two scFvs derived from one anti-BCMA antibody and one anti-CD3 antibody respectively. Preclinical studies have proved that AMG420 could induce BCMA-dependent T cell activation and MM cell apoptosis. In vivo experiments also proved that AMG420 could consume BCMA + MM cells in cynomolgus monkey models [107]. The first in-human dose-escalation trial has been executed to evaluate the efficacy and safety of AMG420 in R/R MM patients. The dose ranged from 0.2 µg/ d to 800 µg/ d and the drug was administered by continuous intravenous infusion. This study demonstrated that the dose of 800 µg/d could not be tolerated by patients. But the dose of 400 µg/d was appropriate. At the dose of 400 µg/d, the response rate and CR rate reached 70% (7/10) and 50% (5/10), respectively. In this trial, the overall response rate was 31% (13/42).

The adverse effects of AMG 420 in this clinical trial were acceptable. 38% of 42 R/R MM patients suffered CRS and one of them reached grade 3. Severe central nervous system adverse events were not observed. Common severe adverse events included infections (14/42) and polyneuropathy (2/42). Compared with blinatumomab, the adverse event rates of AMG 420 were lower, which might be attributed to the distinctions between CD19 and BCMA and the differences between diseases [108].

### AMG 701

AMG 420 should be administered by persistent intravenous infusion to maintain its blood concentration. This character has restricted the application of AMG 420. Another BCMA/CD3 BiTE with a longer half-life is required. And AMG 701 meets the demand because the Fc domain enables it to have a longer half-life. An experiment in vitro demonstrated that AMG 701 enabled T cells to attack tumor cells more efficiently. Animal experiments demonstrated that AMG 701 restricted MM tumor growth in mouse models and resulted in the consumption of plasma cells in cynomolgus monkey models. The combination therapy with PD-1 blockers resulted in synthetic effects [109]. Immunomodulatory imide drugs including lenalidomide and pomalidomide could make AMG 701 more potent in xenograft models [110]. Phase I clinical trials of AMG 701 in patients with R/R MM are being conducted.

### GPRC5D/CD3

GPRC5D is a seven transmembrane protein expressed on MM cells specifically [111]. It is predictive of the poor outcomes of MM patients. High expression of GPRC5D is associated with translocation t (4;14) (p16; q32), which is also a poor predictive factor [112, 113]. Compared with BCMA, GPRC5D is fixed more firmly to the membrane because of its seven transmembrane structure. So the protein is less likely to shed from the membrane and T cells can also be bound to tumor cells in a tighter way, which promotes the cytotoxicity of BsAbs [114]. GPRC5D is a promising target for treating MM and anti-GPRC5D/CD3 BsAbs are being developed.

Talquetamab, also known as GPRC5D T cell–redirecting antibody, recruits T cells to tumor cells and activate T cells. It is produced on the base of DuoBody technology. This drug has shown anti-tumor activity in xenograft mouse models. However, we still lack information about the safety of this drug [111]. A clinical trial focusing on the safety and recommended phase II dose of talquetamab in R/R MM patients is ongoing (NCT03399799).

### CD38/CD3

CD38 is a transmembrane protein which consists of an intracellular domain, a transmembrane helix, and a larger extracellular domain [115]. CD38 is selectively expressed on MM cells, thus this molecule enables researchers to distinguish MM cells from normal cells [116]. Daratumumab, a CD38 monoclonal antibody, has been demonstrated to be efficient and safe in MM patients [117]. Researchers have developed several anti-CD38/CD3 BsAbs including AMG 424 and Bi38-3.

AMG 424, a bispecific T cell-recruiting antibody based on XmAb platform, was selected from a group of anti-CD38/CD3 bispecific drugs because it could delete target cells efficiently without causing severe CRS. AMG 424 can delete CD38 + tumor cells at low concentration and it is expected to be potent in relapsed patients after daratumumab therapy. Although T cells expressing CD38 might be attacked by AMG 424, this side effect is acceptable because the advantages outweigh the disadvantages [118]. Another anti-CD38/CD3 BiTE, Bi38-3, has also shown its efficacy. Bi38-3 could restrict the growth of xenograft MM tumor in mouse models. Compared with AMG 424, Bi38-3 has fewer adverse effects on normal cells [119].

### FCRL5/CD3

FCRL5 is expressed on the sub-groups of B cells. Anti-FcRH5/CD3 BsAbs can recruit T cells to attack plasma cells and MM cells [120]. The extracellular domain of FcRH5 is large, which interferes with the crosstalks between T cells and tumor cells. The anti-FcRH5/CD3 T cell-dependent bispecific antibody (TDB) developed on the base of KiH technique has shown efficacy in attacking FCRL5 + MM cells. In mouse models and cynomolgus monkey models, anti-FcRH5/CD3 TDB could restrict the growth of xenograft MM cells. The combination therapy with PD-1/PD-L1 blockers could improve the potency of anti-FcRH5/CD3 TDB. The half-life of anti-FcRH5/CD3 TDB is long, thus it should be administered intermittently. Release of cytokines usually happened immediately after administration, but it was not severe nor persistent [121].

### Non-Hodgkin lymphoma

Non-Hodgkin lymphoma (NHL) includes all lymphomas except for Hodgkin lymphomas. There are huge differences between aggressive lymphomas and indolent lymphomas. It has been proved that the interaction between lymphomas and the immune system plays a key role in the development of lymphomas [122–125]. Antibodies which regulate the immune system have been proved to be efficient in deleting NHL cells [126]. BsAbs which target NHL cells and T cells simultaneously can form synapses between targeted cells and induce cytotoxicity of T cells [127]. Ideal targets of BsAbs include CD19, CD20, and CD47. The Efficacy of blinatumomab in NHL patients has been illustrated in the efficacy of blinatumomab part. Although BsAbs have shown impressive efficacy in NHL therapy, they are not satisfying in CLL therapy. CLL is a highly heterogeneous disease with acquired immune dysfunction. Prognostic biomarkers and risk scoring systems play important roles in guiding CLL treatment decisions [128]. The strong immunomodulatory effect of CLL causes low response rates to immunotherapy strategies. The emerging antibody products with experimental evidence, such as OTSSP167, mosunetuzumab, and blinatumomab, have offered hope to CLL patients[129, 130].

### CD20/CD3

CD20 is a kind of unglycosylated phosphoprotein. It is specifically expressed on B cells and is an ideal target for the treatment of B cell malignancies. Rituximab, an anti-CD20 monoclonal antibody, has been widely used in the clinical treatment of NHL [131]. However, there are a fraction of patients who have no response to rituximab therapy [132]. Anti-CD20/CD3 BsAbs which can mediate the interaction between T cells and CD20 + tumor cells are a viable option for the treatment of R/R NHL.

### REGN1979

REGN1979, an anti-CD20/CD3 BsAb with the natural IgG-like structure, is being evaluated among R/R B-NHL patients in a phase I clinical trial. At the doses of 5-27 mg, the ORR in R/R FL (Gr 1–3a) patients reached 100% and the ORR in R/R DLBCL patients was 40%. In R/R DLBCL patients treated with REGN1979 and REGN2810, the response rate rose with the dose of REGN1979. At doses of 5-12 mg, the response rate in R/R DLBCL patients was 18% (2/11). At doses of 18-40 mg, the rate was 55% (6/11). When the dose increased to 80 mg, the response rate was 100% (2/2). But the higher dose was related to the higher rate of CRS.

### CD20-TCB

CD20-TCB (RG6026) is an anti-CD20/CD3 BsAb with two CD20 binding sites and one CD3 binding site. This unique 2:1 Crossmab format promotes the function of T cells efficiently. Compared with other BsAbs, CD20-TCB has a longer half-life and higher potency. A phase I study has proved that the ORR in R/R NHL patients was 38% when the dose reached 300 µg or higher. The common adverse drug reactions included neutropenia and CRS. In order to improve the safety of CD20-TCB, researchers have combined CD20-TCB with obinutuzumab pretreatment and this therapeutic regimen is being investigated [133].

### Mosunetuzumab

Mosunetuzumab is an anti-CD20/CD3 BsAb with a full-length humanized structure and the production of this antibody is supported by KiH technology [134]. This drug, also known as CD20-TDB, can recruit T cells to attack xenograft CLL cells in mouse models [135]. In a phase I study which included R/R NHL patients, mosunetuzumab has shown promising potency. The ORR in indolent NHL patients and aggressive NHL patients were 64.1% and 34.7%, respectively.

### CD47

CD47 is composed of an extracellular V-set IgSF domain, a transmembrane-spanning domain and a selectively spliced cytoplasmic tail [136]. CD47 is widely expressed in normal human cells, but it is specifically overexpressed in NHL cells [137]. The interaction between CD47 and signal regulatory protein alpha (SIRPα) plays an important role in the progression of NHL. CD47 interacts with SIRPα and sends a "don't eat me" signal to macrophages, thus inhibiting the macrophage phagocytosis of tumor cells [123]. Therefore, several therapeutic strategies have been developed to block the CD47-SIRPα signaling pathway. These drugs include TTI-621, HU5F9-G4, ALX-148, and CC-90002 [138]. CD20-CD47SL, a BsAb targeting CD47 and CD20, was investigated by Piccione et al. for the treatment of NHL. CD20-CD47SL was demonstrated to eliminate detectable NHL cells in mouse models. Compared with anti-CD20 monotherapy and anti-CD47 monotherapy, CD20-CD47SL significantly prolonged the survival of mice [139]. BsAbs targeting CD47 belong to the rapidly advancing frontier and more novel antibodies are being developed.

## Hodgkin lymphoma

Hodgkin lymphoma (HL) is a sort of B cell lymphoid hematopoietic malignancy. HL is characterized by CD30 + Reed-Sternberg cells surrounded by the immunoinhibitory microenvironment consisting of lymphocytes, eosinophils, plasma cells, and neutrophils [140]. Therefore, there are immune barriers to the success of immunotherapy for HL. To optimize the treatment of HL, one anti-CD30 antibody–drug conjugate (brentuximab vedotin) and two immune checkpoint inhibitors (nivolumab and pembrolizumab) have been developed and these drugs can significantly improve the prognosis of R/R HL [141]. BsAbs targeting CD30 are also promising for the treatment of HL. AFM13 is a bispecific NK-cell engager that specifically targets CD30 + HL cells and CD16A + NK cells. It can activate NK cells and induce tumor cell apoptosis effectively [142]. The safety and tolerability of AFM13 in HL treatment has been demonstrated in a phase I trial implemented by Achim Rothe et al. The overall effective rate of AFM13 in R/R HL patients was as high as 61.5% [143]. More studies focusing on AFM13 in HL patients are being conducted to improve the efficacy of the treatment.

### Myelodysplastic syndromes

Myelodysplastic syndromes (MDS) are a group of highly heterogeneous hematologic malignancies originating from hematopoietic stem cells. They are characterized by ineffective intramedullary hematopoiesis which results in myeloid hyperplasia and peripheral blood cytopenia [144]. The biological and clinical manifestations of MDS vary widely but the treatment options are limited, including follow-up observation, erythropoietic stimulants therapy, immunosuppressive therapy, demethylation therapy, and HSCT [145]. The survival rate of patients with MDS is low. About 25% of high-risk and very high-risk MDS develop to AML within one year [146]. Therefore, it is necessary to develop novel and effective therapies to treat patients with MDS.

It has been demonstrated that CD123 is overexpressed in the bone marrow of MDS patients, which indicates that CD123 is an ideal biological marker and therapeutic target of MDS [147]. Several antibodies targeting CD123, such as KHK2823 and IMGN632, are in clinical trials (NCT02181699 and NCT03386513). MGD006, a DART that targets CD123 and CD3, is currently under a phase I clinical trial.

There is evidence that the proportion of CD33 + cells increases in the bone marrow of MDS patients and bispecific T cell-recruiting antibodies targeting CD33 are being developed. AMV564, a tetravalent anti-CD33/CD3 BsAb, has been proved to be efficient in treating MDS. It could delete MDS cells dose-dependently and restore immune homeostasis in vitro. The phase I clinical trial of AMV564 in MDS patients is underway [148, 149].

## Conclusions

BiTEs have shown great potency in treating patients with hematologic malignancies. By binding T cells and tumor cells through the specific structure, bispecific T cell engagers enhance tumor lysis effectually and provide relapse/refractory patients with feasible options regardless of mutations or T cell dysfunction. Further advances in molecular structures, dosing regimens and combination therapies can help to improve the efficacy and safety of BiTEs. Innovative platforms enable the production of novel bispecific T cell-recruiting antibodies with higher affinity, greater flexibility and longer half-lives. The efficacy and toxicity of emerging new drugs are evaluated in clinical trials. Deeper investigation of combination therapy with PD-1/PD-L1 blockers is expected to prevent tumor escape efficiently. We are optimistic that such knowledge will facilitate the evolution of anti-tumor strategies focusing on BiTEs.

## Data Availability

Not applicable.

## Abbreviations

ADCC: Antibody-dependent cell-mediated cytotoxicity
ALL: Acute lymphocytic leukemia
allo-HSCT: Allogeneic hematopoietic stem cell transplantation
AML: Acute myeloid leukemia
BCMA: B cell maturation antigen
BiTE: Bispecific T cell engager
B-ALL: B cell progenitor acute lymphoblastic leukemia
CAR-T: Chimeric antigen receptor T cell
CD: Cluster of differentiation
CDC: Complement-dependent cytotoxicity
C_H_: Constant region of the heavy chain
C_L_: Constant region of the light chain
CLEC12A: C-type lectin domain family 12 member A
CLL: Chronic lymphocytic leukemia
CLL-1: C-type lectin like molecule-1
CR: Complete remission
CRh: Complete remission with partial hematologic recovery in peripheral blood
CRP: C-reactive protein
CRS: Cytokine release syndrome
DART: Dual-affinity retargeting antibody
DLT: Dose-limiting toxicities
DLBCL: Diffuse large B cell lymphoma
FCRL5: Fc receptor-like 5
FDA: United States Food and Drug Administration
FISH: Fluorescence in situ hybridization
FLT3: FMS-like tyrosine kinase 3
Fv: Variable fragment
Fc: Fragment crystallizable
GM-CSF: Granulocyte macrophage colony-stimulating factor
GPRC5D: G protein-coupled receptor 5D
HSCT: Hematopoietic stem-cell transplantation
HL: Hodgkin lymphoma
HLH: Hemophagocytic lymphohistiocytosis
IFN: Interferon
IgG: Immunoglobulin G
IGHV: Immunoglobulin heavy chain variable region
IL-3Rα: Interleukin 3 receptor alpha chain
IMiD: Immunomodulatory imide drugs
IL: Interleukin
KiH: Knobs-into-holes
MDS: Myelodysplastic syndromes
MDSC: Myeloid-derived suppressor cell
MICL: Myeloid inhibitory C‐type lectin‐like receptor
MM: Multiple myeloma
MP: Melphalan plus prednisone
MRD: Minimal residual disease
MTD: Maximum tolerated dose
NHL: Non-Hodgkin lymphoma
NK: Natural killer
OS: Overall survival
ORR: Objective response rate
PBMC: Peripheral blood mononuclear cells
PD-1: Programmed cell death protein 1
PD-L1: Programmed death-ligand 1
PFS: Progression-free survival
Ph: Philadelphia chromosome
R/R: Refractory or relapsed
s-BCMA: Serum B cell maturation antigen
scFv: Single-chain variable fragment
SIRPα: Signal regulatory protein alpha
TandAb: Tandem Diabody
TCB: T cell bispecific antibody
TCR: T cell receptor
TDB: T cell-dependent bispecific antibody
TFS: Treatment-free survival
TKIs: Tyrosine kinase inhibitors
V_H_: Variable region of the heavy chain
V_L_: Variable region of the light chain
WT1: Wilms' tumor gene 1
